# Coronary Bypass Surgery in a 105-Year-Old Patient with Cardiopulmonary Bypass

**DOI:** 10.1155/2010/725173

**Published:** 2010-06-14

**Authors:** Temucin Noyan Ogus, Filiz Erdim, Ozer Selimoglu, Fatih Tekiner, Murat Ugurlucan

**Affiliations:** ^1^Bahcelievler Medical Park Hospital, Cardiovascular Surgery Clinic, Istanbul, Turkey; ^2^Duzce Ataturk State Hospital, Cardiovascular Surgery Clinic, Duzce, Turkey

## Abstract

Coronary artery bypass grafting is one of the routine daily surgical procedures in the current era. Parallel to the increasing life expectancy, cardiac surgery is commonly performed in octogenarians. However, literature consists of only seldom reports of coronary artery bypass grafting in patients above 90 years of age. In this report, we present our management strategy in a 105-year-old patient who underwent coronary artery bypass grafting at our institution.

## 1. Introduction

Since the average life expectancy increases, there is a need for coronary bypass surgery for advanced-age patients and there are also some operative risks depending on the age of the patient [1]. Refinements in myocardial protection, minimally invasive cardiac surgery, and postoperative care have all contributed to these improved outcomes. However, in contrast to a significant decrease in mortality rates, the presence of multiple comorbid risk factors still places these patients at higher risk for developing postoperative complications which in turn increases the morbidity and mortality rates of each cardiovascular surgeon and hospital costs. 

We made a search in the literature and we have not been able to find any study in which coronary artery bypass grafting (CABG) is applied for the patients over the age of 100. Therefore we analyzed our case with the studies conducted on octogenarians [2]. In this case, our approach towards a 105-year-old case who has undergone CABG with cardiopulmonary bypass (CPB) is presented. 

## 2. Case Report

A 105-year-old male patient, who has a known cardiac disease for almost 5 years, was admitted to our cardiology clinics when his complaints aggravated in the last week. Ejection fraction was 45% in echocardiography. Coronary angiography findings are as follows: 95% of stenosis in the left anterior descending (LAD) ostial, circumflex (Cx) diffused mid area with plaques and lesions by 80%, and 70%–80% of stenosis in right coronary artery (RCA). In the physical examination, arterial blood pressure was found to be 100/60 mmHg, and crepitation was also detected in the basal segments of the lungs after listening the chest. Blood analysis findings are as follows: urea 87 mg/dL, creatinine 1.7 mg/dL. The patient had pain at rest and 2-3 mm of elevation was seen at the anterior derivations, which was seen at the ECG. Our case had unstable pain, coronary lesions were not suitable for PTCA procedure, and there were preshock findings. Therefore we decided to go for semi-urgent CABG. The son of the patient was a doctor, which helped us to explain the risks of the surgery. Classical median sternotomy was performed for the surgery. Ascending aorta was found to be calcified after exposing the pericardium. We decided to use the previously published our method for calcified aorta patients [3]. 

Cardiopulmonary bypass was achieved with right axillary artery and right atrial standard “two-stage” venous cannulation. We started to cool down the temperature of the patient to 28°C. The nonpulsatile flow rate was adjusted to 2.4 L/min/m^2^ to maintain a mean arterial blood pressure of 65 mmHg. The distal anastomoses were performed on LAD and RCA posterior descending—Cx major obtuse branch sequential with saphenous vein grafts under ventricular fibrillation without cross-clamping. Proximal anastomoses were performed on plaque-free area of the ascending aorta by reducing the pump flow to a mean arterial pressure of 20–25 mmHg. During this low flow, we placed ice bags around the head of the patient and trendelenburg position is given to the table. Each proximal anastomosis lasted around 3 minutes and between each proximal anastomosis flow is increased to normal, that is, 65 mmHg at 28°C. Then the temperature was increased and CPB lasted 92 minutes. The operation was finished by classical method and the patient was taken to the ICU. Hydration was continued due to gradual reduction of urea creatinine. No neurologic event was observed postoperatively. The patient was discharged the hospital on the 18th day postoperatively. It was found that the patient had no problem at his policlinic controls at the fourth week. 

## 3. Discussion

Many clinical studies have recently published researches on the results of open cardiac surgery on elderly population, preoperative risk factors, medium and long-term survival, and quality of life [4, 5].

Fruitman et al. reported a 7.9% of mortality rates in octogenarians who underwent a cardiac operation [6]. Previous studies have reported a high incidence of postoperative complications in octogenarians [7]. Basaran et al. reported that the most common complications observed at the early postoperative period included prolonged ventilation time, transient renal dysfunction, atrial fibrillation, sternal dehiscence, respiratory system, and leg wound infections [8]. Additionally neurocognitive impairment is another major complication of open-heart surgery especially in this age-group patient [9, 10]. We did not detect postoperative permanent arrhythmia or neurologic deficit in our case. Preoperative mild renal failure was recovered by postoperative medical treatment. However, the postoperative hospital stay was 18 days, which may be considered as long when compared to a younger patient undergoing cardiac surgery.

Several previous studies have demonstrated the beneficial impacts of off-pump CABG surgery on octogenarians [11]. Another option in advanced-age patients may be hybrid revascularization which may be performed using on-pump or off-pump techniques in suitable cases. In this method, both conventional angioplasty and surgical revascularization are performed concomitantly and consecutively. Although hybrid revascularization seems beneficial, it carries increased risk of bleeding due to anticoagulant or antiaggregant agents used during both methods [12, 13]. We planned for full revascularization due to unstable angina pectoris of the case. We did not find off-pump surgery or hybrid revascularization appropriate since we have detected calcified aorta in our case. 

Another issue is the usage of the internal thoracic artery for revascularization. As in this particular patient as well as other cases with sclerosed ascending aorta, this particular graft has the advantage of not requiring a proximal anastomosis. Moreover, if multiple grafting is considered, the other grafts may be anastomosed onto the internal thoracic artery. However, there are several consequences of the usage of this graft. First of all, during the harvesting, although it is not necessary, intentionally or accidentally the pleura may be opened and it is associated with respiratory compromise after the surgery. Additionally, internal thoracic arteries provide the blood supply for the sternum [14]. Despite the benefits of the internal thoracic artery, we did not prefer this graft in our case in order to minimize respiratory complications, risk of sternal dehiscence, infection, and bleeding and provide short operation. 

One of the major issues in advanced-age patients undergoing CPB is neurocognitive impairment and several studies in the literature have linked fast rewarming to an unfavourable neurologic outcome [9, 10]. In this context, the duration of CPB was 92 minutes in our patient which consisted of a long and gradual rewarming phase.

It is well known that palpation of the ascending aorta during surgery may not identify plaque in all situations. In order to clearly document and detect the exact locations of critical plaque formations in the ascending aorta, perioperative epiaortic scanning by ultrasonography with a sterilized probe may be performed [3]. However, due to the lack of the equipment at that time and relative emergent condition of the patient, we relied on careful palpation of the ascending aorta as well as the calcifications seen at the ascending aorta at the preoperative chest roentgenograms and coronary angiography. 

## 4. Conclusion

As in this case presentation, there are several modifications needed for the surgical strategy for advanced-age group since CABG operations are more risky. The data obtained in these cases will be investigated in detail in the larger series to be conducted in the future. 
